# Good Without God: Bioethics and the Sacred

**DOI:** 10.1007/s12115-015-9927-x

**Published:** 2015-09-11

**Authors:** R. De Vries

**Affiliations:** Center for Bioethics and Social Sciences in Medicine, University of Michigan Medical School, 2800 Plymouth Road, Ann Arbor, MI 48109-2800 USA

**Keywords:** Religion and Bioethics, Suffering, Culture and Bioethics

## Abstract

Bioethics traffics in matters moral. As such, bioethics frequently bumps up against religion, offering an ideal arena to examine how the sacred and the secular encounter each other in modern medicine. In this essay I consider two places where bioethics and religion intersect: 1) the response of bioethics to the universal problem of suffering, and 2) the professional proselytizing or “missionizing work” that bioethics does in order to make a place for itself among the professions of the life sciences.

I hate, I despise your religious festivals;

your assemblies are a stench to me.

Even though you bring me burnt offerings and grain offerings,

I will not accept them.

Though you bring choice fellowship offerings,

I will have no regard for them.

Away with the noise of your songs!

I will not listen to the music of your harps.

But let justice roll on like a river, righteousness like a never-failing stream!

Amos 5: 21–24

I have a small poster on the door of my office that offers a concise illustration of the relationship between science and the humanities. It shows a scientist, clad in white lab coat, watching as a very large egg cracks open. His arms are raised above his head in celebration. The text reads: “Science can tell you how to clone a tyrannosaurus rex”. Immediately below is a second image: the same scientist running from the now full-grown, fearsome dinosaur. Here the text reads: “Humanities can tell you why this may be a bad idea”. Perhaps a bit simplistic, but it captures the tension between the sacred and the secular that we find in the modern hospital. The myriad technologies that populate the clinic – all of which are capable of altering the natural processes of birth, illness, and death – create questions about their proper application, questions that cannot be answered by the secular and rational logic that built these technologies.

Where *do* we go for guidance about how to use the technologies of medicine? That depends, in large part, on who the “we” is. If the “we” includes those who agree on a set of moral principles and on the source of those principles, the answer is relatively simple. There may well be disagreement on how to *apply* those agreed-upon principles, but at a minimum the decision about where to begin has been made.

But if the “we” encompasses a collection of folks whose moralities begin in different places and generate a variety of moral principles, agreeing on the right thing to do will be no easy task. Sorting right from wrong in this situation forces us to notice the relationship between cosmology and morality, to ponder the nature of the moral authority – if any – we are willing to submit to. As societies move from religious to scientific cosmologies, moral authority moves from the sacred to the rational. But this move is never complete. Logical and empirical explanations of *why* something is moral or immoral cannot fully encompass questions of *meaning* that are part of ethical decision-making. For example, medical-scientific evidence about the viability of a 20-week-old fetus gives no counsel about whether that fetus is a human being or merely a proto-human, a “product of conception”.

Enter bioethics. Bioethics was born in a context characterized by moral pluralism and shifting ideas about the nature of moral authority; it was, and is, an effort to develop a set of principles and a method for moral decision-making acceptable to all, regardless of one’s religion or ideology. Given its mission to bring a secular morality to medicine and the life sciences -- where ethical questions about the boundaries and meaning of life abound -- bioethics finds itself constantly bumping up against religion. These encounters are challenging because they highlight an essential tension in the precepts of bioethics. Foundational to the field is “respect for persons”, a precept that demands that religious beliefs not be dismissed as merely collections of irrational myths. At the same time, the bioethical precept of universalism makes it impossible to include religion as a serious element in moral decision-making. As a consequence, bioethics compartmentalizes religion. Yes, religion has some *limited* moral legitimacy, appropriate for certain groups and individuals, but it has no claim on broader moral theory. Thus religion is ghettoized: we have, for example, Catholic bioethics, Jewish bioethics, and Islamic bioethics.

This unique tension makes bioethics an ideal arena to explore how the sacred and the secular encounter each other in modern medicine and medical research. I am particularly interested in two of the many places where bioethics brushes up against religion. The first has to do with the sociology of bioethical knowledge: how bioethics approaches and responds to the universal problem of suffering. The second is “professional proselytizing”: the “missionizing work” that bioethics does in order to make a place for itself among the professions of the life sciences. In both cases I consider how the secular stance of bioethics limits its ability to fully respond to the challenging existential and moral questions that emerge in medicine and medical research.^1^

## Secular Bioethics and the Response to Suffering

While interest in the ethics of medicine is as old as the healing arts themselves, bioethics is, as noted above, a relatively recent field of inquiry and practice. There are varied accounts of the conditions that generated the move from medical ethics to bioethics, but common to all is concern with the unnecessary suffering of research subjects and patients. These histories reference the harmful experiments conducted on humans without their consent during World War II (notably by the Germans, but also by the Japanese and the Americans), the use of poor people and minorities in medical research,^2^^,^^3^ and the tough choices that had to be made when kidney dialysis was new and available only to a few.^4^

It is, in fact, fair to say that bioethics was born because of this deep concern with human suffering. Remarkably, in spite of this foundational disquiet, the field has spent very little intellectual energy on responses to suffering. As the *secular* voice of moral decision making, bioethics has ceded inquiries on the nature and meaning of suffering to religion. Anthropologist Arthur Kleinman comments:

One is surprised to find so many professional ethical volumes in which [the word suffering] does not appear as an entry in the index. Ethical systems that leave the problem of suffering (and related concepts of endurance and courage) to particular theological traditions cannot adequately engage the human core of illness and care.^5^

Two features of bioethics are resposible for this bioethical ignorance of suffering. First, as John Evans points out in his book *Playing God*, the field has moved from “thick” to “thin” bioethics.^6^ In the early days of the field, bioethicists were concerned with “thick”, substantive questions about the meaning of human life and the effect of new technologies on what it means to be human. The search for answers to these questions drew on theological and philosophical insights. As the field developed, the questions that interested bioethicists became much “thinner” and more formal. No longer was there a concern with *ends*, the interest shifted to *means* – that is, how to design guidelines and regulations that would protect patients and research subjects. Bioethicists became consultants, educators, and guides seeking bureaucratic solutions for bioethical problems. This new “thin” approach provides no space to explore the meaning of suffering. Contemporary bioethics has nothing to say when those who suffer need help in making sense of their experience.

A second reason that bioethics gave up on suffering is that the field has become closely associated with medicine. Kleinman observes:

Like biomedicine, bioethics begins with professional definitions of pathology. The experience of illness is made over, through the application of ethical abstractions into a contextless philosophical construct that is every bit as professionally centered and divorced from patients’ suffering as is the biomedical construction of disease pathology.^7^

He goes on to say:

… [the] standard version of bioethics shares yet another biomedical bias, the rejection of teleology. Biomedicine banishes the concepts of purpose and ultimate meaning to religion; yet most patients and practitioners struggle to make sense of illness with respect to great cultural codes that offer coherent interpretations of experience.^8^

By giving up on meaning and teleology, bioethics misses what we might call ‘the paradox of suffering’. Cassell has defined this paradox well:

…suffering also reveals to the sufferer a greater depth of human experience and meaning. After the experience of suffering, the person is led to a richer understanding of the meaning of being human, a greater concern for the suffering of others, and away from the superficialities that too often characterize daily existence.^9^

What does bioethics lose when it ignores the paradox of suffering? In his book, *Lost in the Cosmos*: *The Last Self*-*Help Book*, American novelist and essayist, Walker Percy, offers an answer. In one of several anecdotes, he describes an ordinary morning when a businessman goes out to retrieve the morning paper. He is suffering from anxiety and concerned about a presentation he must make later in the day, and in a bizzare twist, an insane young man drives by and shoots him with a .22 caliber pistol. The wound is not fatal, but as this businessman waits for the ambulance, he notices how well the dogwood he planted 10 years ago is doing; in the hospital he jokes with the doctors, quoting Churchill who said: “Nothing makes a man feel better than to be shot without effect.” Percy then asks his readers, “Is this occurrence:

- Unrelievedly bad news? It is not good to get shot. One could die of it.
- Putatively bad news but secretly good? The incident somehow dispenses you. The single irrational act of a madman changes the entire state of your life in an instant – from that of an anxious worried businessman in danger of losing a big account, to that of an innocent victim, not only not guilty, but also unfailed…”^10^

Percy uses this story to reveal one of the possibilities of suffering: Suffering takes us out of our ordinary lives and makes us aware of things going on about us that do not normally get noticed.

Given its thin and non-teleological approach to affairs medical, bioethics is unable to provide this type of insight to those who suffer. Bioethics consultations are limited to resolving questions about “morally appropriate” care. Efforts to make sense of the illness experience are necessarily excluded.

Looking more deeply into the paradox of suffering, we discover that the experience opens up three distinct possibilities. First, suffering *creates community*. It is in suffering that we discover what we share with our fellow humans. Neiderauer makes this observation:

In human suffering the believer sees the grounds of our common humanity, recognizing that it is through suffering, above all, that human beings are stirred to the love of one another… 11

In his book, *The Wounded Healer*, Henri Nouwen picks up on this theme. He points out that being wounded allows the healer – be that a minister, a physician, or a bioethicist – to show true hospitality. He says there is more to being a wounded healer than just spiritual exhibitionism, or the sharing of superficial personal pains. He sees in the wounds that the healer brings with him or her “a constant willingness to see one’s own pain and suffering as rising from the depths of the human condition which we all share.” In this way, suffering frees us from ourselves, from our illusions of immortality and illusions of wholeness. When we are freed from these illusions and from ourselves we can truly welcome the “other” and be authentically hospitable.^12^

Second, suffering also can lead to *resistance*. The sufferer is separated from the world that has pushed this person in directions that run counter to true human need. Kleinman explains:

Take, for example, consumer society. I can’t tell you how many patients I’ve sat with who had terrible cancers or other life-threatening problems who’ve said to me, “You know, one of the things about this disease is it’s really made me rethink how I live my life.” That’s a kind of protest, a resistance to the way the world is that comes out of that encounter.^13^

James Hillman agrees. He believes that suffering creates the possibility of revolution. He says, “The real revolution in our society begins with a person who can stand with his own depression, because then you say, ‘No!’ to the whole manic situation of modern society – over-consumption, over-activity, travel.”^14^

Third, suffering can be regarded as a *quest*. Arthur Frank, who has written much about suffering from the point of view of sociology, finds three ways that people approach suffering.^15^ One is to stay put in their suffering, which he defines as chaos -- “living among the broken integrity of the person.” A second approach is to go backward, that is, to reconsider your life, thinking about events that you now regret, thinking about ways to repair those regrets, to engage in restitution and undo the wrongs you have done. The third response to suffering is to go forward and to see suffering as a quest, opening up new possibilities in life, taking people to new places where they had not been before.

This point of view about suffering is well illustrated in Flannery O’Connor’s short story, “A Good Man is Hard to Find.” The story begins as a family is preparing for a trip to Florida. Things are typically hectic, complicated by the fact that “grandmother”, the classic mother-in-law, does not want to go. In an effort to get her way, she picks up the paper and reads aloud about the “Misfit”, who has just escaped from the Federal Pen and is “headed toward Florida”. The Misfit is a terrible man who kills without mercy -- a one-man machine of pointless suffering. If you know the work of O’Connor, devout Catholic from the American South, you know that mention of the trip to Florida and an escaped criminal guarantees a fateful meeting.

The family finally gets out the door and the grandmother continues to nag, insisting they take a detour to see an old plantation house with a fascinating ‘secret panel’. Dad was not inclined, but the children took up the cause, screaming that they wanted to see the secret panel. Being a good father, he gives in and heads off down the dirt road that the grandmother is sure will take them to the house with the secret panel.

You can guess the rest. The family cat – Pitty Sing – escapes from her basket and startles the dad who rolls the car into a ditch. As they get out of the car, checking themselves for injury, who should appear from the woods? The details are classic O’Connor. The Misfit and his accomplices dispatch dad, mom, and the kids, leaving the grandmother to plead for her life:

The misfit’s voice seemed about to crack and the grandmother’s head cleared for an instant. She saw the man’s face twisted close to her own as if he were going to cry and she murmured, “Why you’re one of my babies. You’re one of my own children!” She reached out and touched him on the shoulder. The misfit sprang back as if a snake had bitten him and shot her three times through the chest. Then he put his gun down on the ground and took off his glasses and began to clean them.

One of his accomplices comments: “She sure was a talker, wasn’t she?” The misfit replies: “She would of been a good woman, if it had been somebody there to shoot her every minute of her life.”^16^ Suffering can be seen as the experience that is there to shoot us every minute of our lives.

Cassell summarizes this for us:

Both on a religious and a secular basis, it is not unusual for suffering persons to believe that their suffering is a form of selfless service to others. Through the acquisition of meaning in this fashion, the suffering is alleviated.^17^

The commitment of bioethics to be the secular approach to moral decision-making has weakened the ability of the field to respond to (inevitable) suffering and to fully appreciate the ethical aspects of illness and its treatment. What would bioethics look like if it was not cocooned in its secular space? Integration with religion would allow bioethics to move from the realm of the extraordinary to the ordinary, to consider the inevitable, everyday, ordinary problems of living and to see the need to see the need to respond to those problems as part of the moral responsibility of the medical community. In overlooking the universality of suffering, bioethics misses the opportunity to engage in a deeper understanding of who we are as humans – an understanding essential to moral foundations of medicine. Exploring suffering can tell us something about the nature of persons, the relationship between persons and their bodies, the goals of medicine, the relationship between persons and their communities, and the place of the spirit in the lives of individuals.

## Spreading the Word: the “Good News” of Bioethics

As a secular, rational endeavor, bioethics resists the religious. But, interestingly, in its efforts to establish its place in medicine and the life sciences, bioethics calls on proselytizing methods commonly associated with religious missionizing. Like Christian missionaries who left Europe and North America to witness to peoples in other lands, bioethicists are now bringing the gospel – the “good news” – to those in low and middle income countries.

The Christian gospel is the New Testament story of salvation found in the birth, death, and resurrection of Jesus Christ. The gospel of bioethics is “good clinical practice”, the Belmont Report,^18^ and the Declaration of Helsinki.^19^ Both missionaries and bioethicists may object to this comparison. Missionaries will point out that they are devoted to a *spiritual* task, not to the secular work of developing regulations and guidelines for the practice of medicine and medical research. For their part, bioethicists will likely resent being described with a term associated—in their minds, at least—with those who destroyed local cultures and paved the way for colonial abuses. These objections are, of course, based on stereotypes. Not all missionaries are tools of imperialistic nations, and most do more than care for the souls of those they minister to. And while it is true that bioethicists help to create and write regulations, their ultimate goal is to protect patients and research subjects from harm and exploitation.

Nevertheless, the metaphor of missionary work is useful for understanding how bioethics has insinuated itself in the developing world, making visible interesting similarities in the work of missionaries and bioethicists. For instance, it is clear that bioethicists have followed—consciously or unconsciously—one important example from the missionary movement. Like missionaries before them, bioethicists are shifting from “imported” to “indigenous” evangelization.^20^ Beginning in the mid-nineteenth century and throughout the twentieth century, missionaries faced resistance from their host countries. In some places, most notably China, missionaries were expelled; in other places, missionaries were increasingly regarded as colonialists. Mission organizations responded to this turn of events with the notion of “indigenization.” No longer would missionaries from the West be exported to other countries. Instead, citizens from those countries would be brought to the West and trained to situate the missionary message in the local culture. This meant translating the gospel into local languages, using local organizational forms in the creation of churches and adapting local customs to the teaching of the gospel. Over time, indigenization came to be called “contextualization”, and it was described as an effort to protect, and be relevant in, local culture.

Although they do not use the term, those in the West who wish to bring the benefit of bioethics to the developing world have seen the value of indigenization. Indigenization is a solution to what Solomon describes as the “export problem” of Western bioethics—a problem that is unavoidable when bioethics, a creation of Western culture, collides with the systems of ethics found in local, non-Western cultures.^21^ Pursuing the indigenization solution, bioethicists from the developing world are currently being trained in the United States (via the Fogarty International Center of the National Institutes of Health^22^), Europe (via the Erasmus Mundus Masters program in bioethics^23^), and the United Kingdom (via The Wellcome Trust^24^). Having learned the language and logic of Western bioethics, trainees return to their home countries to spread the “gospel”.

What do we know about the “success” of the indigenization of bioethics? Research addressing and/or evaluating these specific training programs has been scant, and the descriptions and evaluations that do exist tend to be programmatic (i.e., “Did we meet the goals of the funder?”) rather than critical and reflective (“Have our bioethics programs helped local norms and values to be realized?”).

### From Noble Intent to Unwitting Harm

For the most part, the desire to spread the gospel, Christian or bioethical, begins with noble intent—the goal is to bring the benefits of developments in one part of the world to another part of the world where those benefits are not experienced or understood. Those benefits may be eschatological or existential, but in either case, the motivation is to proffer aid and share lessons learned. But, as we have seen in some of the transactions between missionaries/bioethicists and the people they serve, noble intent is not sufficient to bring good results. An imbalance in power between would-be helpers and those to be helped creates a one-way flow of influence from “missionaries” to “locals” that not only diminishes the possibility of mutual enrichment, but also creates the possibility of unwitting harm.

An evaluation of a research ethics training workshop at a Nigerian university implicitly illustrates the problem of one-way flow of influence. The authors begin their report by noting that “training in research ethics affords scientists, especially those from developing countries, the opportunity to contribute to ever increasing international debates on ethical issues. . .” Indeed, “international debates on ethical issues” *should* be informed by insights of those in the developing world. But just a few pages later, we learn what the program actually accomplished: “Post-training improvements were found in participants’ knowledge of the principles of research, the application of these principles, the international regulations, and the operations of an IRB”.^25^ Measured by its own evaluation metric, this program was focused on teaching Nigerians the wisdom of Western bioethics (“principles, the international regulations, and the operations of an IRB”), not on seeking wisdom from the traditions of Nigeria.

Arguing from a natural law perspective, Boyle points out:

…fragmentation of the pursuits of health around the world implies that no authority within any health care or biomedical community such as a medical association or expert group [can] qualify as having global bioethical authority. . . [U]ntil the world is much more integrated and unified, there will be no properly bioethical legislature or Supreme Court for the whole world.^26^

We know that religious, legal, psychological, historical, and ethical differences have an impact on bioethical views both within and between countries^27^ but this seemingly obvious fact gets lost in many ethics training programs in developing countries. Benatar, a bioethicist from South Africa, chides those from the West who would “improve” the ethics of countries in the developing world:

What should be avoided is the previous colonial mentality of wanting to study and improve others while oblivious of the need to address the more sophisticated and covert faults of Western researchers’ own societies. The desire to improve the behavior of others should also be associated with awareness that one’s own exemplary moral behavior might be more effective in promoting ethical behavior and respect for human rights than […] attempts to change the cultural attitudes of others while neglecting our own adverse cultural attitudes.^28^

### The Inside View of Indigenization: Bioethicists-in-Training Speak

Interviews with 21 trainees at a European-based bioethics program, described elsewhere,^29^ shed light on several important things about efforts to indigenize bioethics. Here I briefly review three of these: (1) problems with the sources of, and models of, ethical reasoning; (2) a lack of fit between the ethical issues taught in classes and the ethical problems in the students’ home countries; and (3) the motivation(s) for establishing Western bioethics in the developing world.

#### Sources and Models of Ethical Reasoning

A majority of the students in this training program came from parts of the world where Christianity was not the dominant religion. And yet, in Europe, reasoning about ethical issues is deeply rooted in the Christian tradition and Christian scholarship of the West. Students were aware of this and felt a degree of disconnect with their own histories. A student from China pointed out:

“…if I want to accept all of the theologies and, too, the methods for the bioethics for Chinese people, it’s a little bit difficult …in China, we have different religions…and very few people believe in God…they are not Christian…so they have no sort of knowledge about Christian history, about Jesus, so you know, a lot of bioethics, methods, and theories came from Jesus…”

Students admitted that much of their own exposure to ethics and bioethics, before their arrival in Europe, came from the West. This is to be expected, given that the overwhelming majority of published knowledge about ethics—in books, reference works, and on the Internet—comes from Europe and North America.

One student pointed out that training in the Western way of bioethics is “interesting,” but the model of ethical reasoning that is almost universally taught—principlism—is not an easy fit in most developing countries.

Based on the well-known four principles—autonomy, beneficence, nonmaleficence, and justice—principlism is easily taught and applied to a wide variety of ethical dilemmas, at least in societies where these principles fit seamlessly with cultural values. It can be argued that the four principles are sufficiently abstract allowing them to float above, and yet account for, the peculiarities of culture. This line of argument suggests that regardless of our cultural differences, we all can agree that nonmaleficence is a good thing. But, of course, we must hasten to add that what you and I call “harm” may vary. And the same can be said of autonomy: in the United States, autonomy is conceived in a radically individualist manner, but in other cultures we can adjust the idea to incorporate more familial and communal ideas of autonomy. In the atomistic United States, a free and independent individual should (must?) determine her care, whereas in more communal societies, autonomous decisions occur in consultation with, or by decision of, recognized authorities.

Ultimately, this argument is specious. Pushed too far in this direction the principles become meaningless. Can we really speak of autonomy if others make a treatment decision for an adult woman? Bioethics students from the developing world saw several problems with the principlist approach they were learning. Not surprisingly, the principle of autonomy was identified as most problematic. Many of the students saw a disjuncture between their cultural values and the individualism implicit in autonomy. A student from a Muslim country noted: “…in [Islam]…justice [is more important] than autonomy…Islam said first your neighborhood…not first yourself.”

Another discussed a Buddhist view:

“…[an author I am reading] argues that in Buddhism you have no concept of autonomy. …She said that the central concept is compassion, and it emphasized paternalism. So for myself, this is what I think for myself, compassionate paternalism, it’s not that bad.”

From India:

“[In rural India] their idea of autonomy is totally different, like they’re not, even for signing our hospital admission sheet, it is not a patient or immediate person, it is…the family [that] was signing for that patient, even for admission, even for taking out of the hospital, it’s not the patient who is signing…we are not thinking the autonomy of the person, we are thinking about the collective autonomy of the whole family.”

#### Ethical Issues Here and There

Training in bioethics typically involves review of ethical theories (described above) and in-depth discussion of critical ethical issues. These discussions offer the opportunity to apply ethical theory to real-life situations, and allow students to practice the move from the theoretical to the applied. What are the ethical issues covered in coursework of students from the developing world? Here are two course descriptions from the curriculum of the students who were interviewed:

- **Ethics of Reproductive Technologies**: The aim of this course is to familiarize participants with an ethical approach of assisted reproduction. The goal is not only to essentially inform the participants about the latest developments and challenges in this area of medicine, but also to help them develop a critical and ethical clarification of this subject. This course works from an interdisciplinary (theological, legal, psychological, medical) perspective.
- **Human Genetics and Medical Technology**: The course aims to educate participants on a range of ethical subjects that currently are the focus of debate in genetics. Teaching will focus on the moral problems generated by the Human Genome Project, as well as the ethical boundaries in the clinical application of new knowledge in, for example, genetic counseling, genetic screening, gene therapy, and cloning. The implications of scientific progress for the image of the human being, as well as for modern culture, will also be studied.

This curriculum is not atypical. Review of the course requirements for those in the Johns Hopkins Fogarty African Bioethics Training Program reveals a few more relevant-sounding titles—for example, there is a “Short Course in International Research Ethics”—but course content is equally skewed toward Western ideas. The description of the International Research Ethics course reads: “Introduce trainees to the major principles and theories of Western bioethics, to U.S. and international guidelines that govern human participants research”.^30^ An evaluation of the program suggests it has been empowering to students but, significantly, also indicates that the training is still too Western-centric, lacking in curricula more appropriate to developing countries.^31^

Students in the European program noticed this lack of fit. At one point, students from the program approached the faculty about making the coursework more relevant to conditions in their home countries. As one student explains, they were rebuffed:

“… would you like to know what was a surprising thing for me? That when we were talking about adjusting this program to the need not just to talk about the [European] situation, but a little bit wider, and people from India were highly interested to talk about HIV…the reaction was, ‘but we are a *European* course.’”

Students also felt that the assigned readings were not aligned with what they would need to know when they returned home. One student talked about the need to learn the basics, even if she did not know “who Levinas is:”

“… for me, it is sometimes too much, too many terms, not the right context, and I will prefer to debate, to talk, to share something like that, because it is the way I learn better, rather than just to have a philosopher who is using a very specific language.”

#### Motivations: Why Teach Western Bioethics to Students from the Developing World?

Given the lack of fit between the content of training programs in bioethics and the bioethical situation and needs of countries in the developing world, it is reasonable to ask “Why do these programs exist?” The answer to that question is not simple. In our search for an answer, we look to both what we learned from students and to campaigns by for-profit organizations marketing their clinical trial services. In her explanation of the difference between medical ethics and bioethics, a student from India reveals an important motivation for the export of Western bioethics (emphasis added):

“Medical ethics was something like the ethics related to clinical care and the doctor, how we should be with patients and all that. That was what we were taught in medical school … But now because research is coming in a big way, especially with a lot of international collaborations, the U.S. ethos of bioethics is coming in a big way.”

This student understands the current situation. As Petryna has pointed out, the number of clinical trials in the developing world has grown markedly over the past 15 years, as pharmaceutical companies search for “naïve bodies” (bodies that are not under the influence of several drugs, as is the case in many Western nations), and more favorable ethical environments.^32^

Students are aware of the need for better bioethics in the developing world. In their comments on the coming of the pharmaceutical industry to their countries, they demonstrate a mix of motives for learning the ways of Western bioethics: to protect the subjects of research but also to encourage economic development. This student from China was typical:

“No, just now, [we have] no formal [research ethics] committees [in China], no, so it’s a big problem. I think I like to come here and learn more knowledge about this field, I want to do some work in this field… at least I can help to organize the bioethical review communities in my universities, for our country…I know doctors, they do some clinical trials, they have no approval…they didn’t do the informed consent…they didn’t have the review…[from] the research review committees, so it’s a big problem, they do some trials, it’s not good.”

She goes on to discuss the director’s enthusiasm for having European-trained bioethicists in China:

“…my director, director of our institute, he’s very good person and he has a lot of ideas for the future development, and when he learned [that] I got [a] scholarship, and of the European community, and I can come to European countries to learn bioethics, he [was] very excited. He said, ‘ahh it’s good, it’s good for you and also for our institutions. I know in Europe the bioethics have a very good…they pay a lot of attention in this field and they have a lot of knowledge in this field and I think you can learn a lot of things there.’”

Similarly, a student from an Eastern European country noted:

“…we have several IRBs registered, just because let’s say our genetics center, probably 7, 8, 10 years ago, became interested to work with some French organization and they, one of the requirements was: ‘where was your IRB?’ So these people started to work on that…”

## Conclusion

Can the essential ideas and goals of a secular bioethics be realized in culturally plural societies? What happens when we are good without God? In its effort to avoid parochialism, bioethics avoids exploring the meaning of illness, suffering, and death. In its effort to protect the subjects of medicine and medical research wherever they may be found, bioethics engages in professional proselytizing, preaching the secular gospel of ethical principles that transcend culture. The good news offered by religions is replaced by a secular good news of rules and regulations.

Bioethicists can succeed in their mission without becoming evangelists if they look more closely at missionary history. McGinnis points this out when he compares the indigenization movement in Christian missions with a similar trend in efforts to promote human rights:

…an essential ingredient in the missionary strategy of evangelization is conspicuously absent in contemporary programs of development, democratization, or peace-building. In particular, the extensive efforts devoted by Protestant missionaries to the translation of their Biblical message into local languages and symbolic repertoires bear little resemblance to efforts to transplant Western ideals of universal human rights, or the institutional templates of democratic governance, first developed in the United States and Western Europe.^33^

The effort to appreciate religious beliefs and local cultural ideals is more than just a sign of respect for the “clients” of bioethics. It also promises new ways of approaching the issues secular bioethicists confront. In their discussion of the value of a “bioethics from below,” Rennie and Mupenda suggest that “bioethics research and scholarship [revolves] around issues that, while fascinating and important, currently affect only a small minority of the world’s population” and argue for a move away from “a ‘90/10’ gap, i.e., a situation where 90 % of discussions on bioethics in the literature and the popular media may revolve around issues affecting 10 % of the world’s population.” They conclude with this interesting comment on the value of *two*-*way communication* between Western bioethics and bioethics in the developing world:

…greater attention to ethical issues arising from biomedical research, clinical practice and public health interventions ‘far away’ might have a positive effect on bioethics ‘closer to home,’ potentially expanding the horizons of the field and enhancing its social relevance.^34^

If they are willing to “thicken” their thinking about ethical problems, secular bioethicists can learn a great deal from the ethical traditions embedded in religions and found in the countries of the developing world. Like Christian missionaries who discovered new things about God by listening to those they hoped to convert, bioethicists will learn new things about ethics by listening to religions and to those in the developing world. Reflecting on the possibility of a global bioethics, Engelhardt explains:

… global bioethics can at best provide a thin moral framework, a space within which individual and moral communities can peaceably pursue divergent understandings of morality and bioethics within limited democracies and within a global market. Such a global bioethics cannot provide a content-full understanding of the right, the good, virtue, or human flourishing. Content will have to be found within particular moral communities and the moralities and bioethics they sustain.^35^

Bioethics has been unwilling to enrich its practice by integrating ideas drawn from religious traditions and from cultures different from the West. As we have seen, there are historical and professional reasons for the move from thick to thin bioethics, but with this move bioethics misses the opportunity to enhance its own moral practices and the moral practice of those who have not given into secularization.

I opened this article with a passage from the Old Testament book of the prophet Amos. When it encounters religious traditions, secular bioethics would do well to keep in mind the voice of Amos, reminding them of the many ways the pursuit of justice is hampered by clinging too tightly to one’s traditions and professional self interests. A more complex and thoroughgoing rapprochement between secular and religious approaches to moral decision-making is overdue.

## Footnotes

1 The following two sections are based on earlier work on these topics published in (De Vries 2011; De Vries and Rott 2011; and De Vries et al. 2011).

2 Skloot 2010.

3 Reverby 2009.

4 Alexander 1962.

5 Kleinman 1997: 50.

6 Evans 2001.

7 Kleinman 1995: 1670.

8 Kleinman 1997: 50.

9 Cassell 2004: 32.

10 Percy 1983: 62–63.

11 Niederauer 2007.

12 Nouwen 1979.

13 Kleinman 2006: 88.

14 Hillman 1983: 12.

15 Frank 1995.

16 O’Connor 1983.

17 Cassell 1995: 1961–1969.

18 Available at: http://www.hhs.gov/ohrp/humansubjects/guidance/belmont.htm.

19 Current version available at: http://www.wma.net/en/30publications/10policies/b3/index.html

20 See http://www.gameo.org/encyclopedia/contents/I54ME.html for a brief description of the indigenization movement in mission work from a Mennonite point of view.

21 Solomon (2006: 337) outlines three different forms of the export problem: temporal, local, and personal.

22 See http://www.fic.nih.gov/programs/training_grants/bioethics/overview.htm.

23 See http://med.kuleuven.be/education/Bioethics/index.html.

24 See http://www.wellcome.ac.uk/Funding/Medical-humanities/Grants/Biomedical-ethics/index.htm.

25 Ajuwon and Kass 2008: 2,8.

26 Boyle 2006: 321.

27 Bayertz 2006: 220–223.

28 Benatar 2005.

29 De Vries et al. 2011.

30 Hyder et al. 2007: 677.

31 Hyder et al. 2007: 675, 682; see also Marshall and Koenig 2004.

32 Petryna 2005.

33 McGinnis 2007: 401.

34 Rennie and Mupenda 2008.

35 Engelhardt 2006: 40.
